# Individuality in coo calls of adult male golden snub-nosed monkeys (*Rhinopithecus roxellana*) living in a multilevel society

**DOI:** 10.1007/s10071-018-1222-y

**Published:** 2018-11-20

**Authors:** Penglai Fan, Ruoshuang Liu, Cyril C. Grueter, Fang Li, Feng Wu, Tianpeng Huang, Hui Yao, Dingzhen Liu, Xuecong Liu

**Affiliations:** 1 Key Laboratory of Biodiversity Science and Ecological Engineering (Ministry of Education), College of Life Sciences, Beijing Normal University, Beijing, 100875 China; 2 School of Environment, Beijing Normal University, Beijing, 100875 China; 3 School of Human Sciences, University of Western Australia, Perth, WA 6001 Australia; 4 Centre for Evolutionary Biology, School of Biological Sciences, University of Western Australia, Perth, WA 6001 Australia; 5 College of Life Sciences, University of Chinese Academy of Sciences, Beijing, 100049 China; 6 Shennongjia National Park, Shennongjia, 442421 Hubei China

**Keywords:** *Rhinopithecus roxellana*, Vocal individuality, Source-filter theory, Multilevel society

## Abstract

Vocal individuality is a prerequisite for individual recognition, especially when visual and chemical cues are not available or effective. Vocalizations encoding information of individual identity have been reported in many social animals and should be particularly adaptive for species living in large and complexly organized societies. Here, we examined the individuality in coo calls of adult male golden snub-nosed monkeys (*Rhinopithecus roxellana*) living in a large and multilevel society. Coo calls are one of the most frequently occurring call types in *R. roxellana* and likely serve as the signals for contact maintenance or advertisement in various contexts including group movement, foraging, and resting. From April to October 2016, April to July 2017, and September to October 2017, we recorded a total of 721 coo calls from six adult males in a provisioned, free-ranging group and one adult male in captivity in Shennongjia National Park, China. We selected 162 high-quality recordings to extract 14 acoustic parameters based on the source-filter theory. Results showed that each of all parameters significantly differed among individuals, while pairwise comparisons failed to detect any parameter that was different between all pairs. Furthermore, a discriminant function analysis indicated that the correct assignment rate was 80.2% (cross-validation: 67.3%), greater than expected by chance (14.3%). In conclusion, we found evidence that coo calls of adult male *R. roxellana* allowed the reliable accuracy of individual discrimination complementarily enhanced by multiple acoustic parameters. The results of our study point to the selective pressures acting on individual discrimination via vocal signals in a highly gregarious forest-living primate.

**Electronic supplementary material:**

The online version of this article (10.1007/s10071-018-1222-y) contains supplementary material, which is available to authorized users.

## Introduction

Vocal cues play an important role in the instant communication of social animals, especially when visual and chemical signals are not available or effective (Kondo and Watanabe 2009; Rendall and Owren 2002). Many animals have evolved a variety of call types that differ acoustically and serve a number of functions, such as maintaining contact (Kondo and Watanabe 2009; Weiss et al. 2001), cultivating social relationships (Bolt and Tennenhouse 2017), and warning each other about predators (Seyfarth et al. 1980; Zuberbühler 2001). However, the acoustic structures of certain call types display graded within-type variation (Soltis et al. 2005). This acoustic variation usually conveys important function-related information about callers, such as group membership (Delgado 2007; Fan et al. 2011), age (Charlton et al. 2009a; Fischer et al. 2004), sex (Charlton et al. 2009a; Ey et al. 2007), body size (Pfefferle and Fischer 2006; Reby and McComb 2003), social rank (Bergman et al. 2003; Fischer et al. 2004), and estrus state (Charlton et al. 2010).

Individual distinctiveness in acoustic features of the same call type has also been reported in many species of social animals (e.g., *Spheniscus demersus*: Favaro et al. 2015; *Pan troglodytes*: Levréro and Mathevon 2013; *Papio hamadryas ursinus*: Rendall 2003; *Dama dama*: Vannoni and McElligott 2007). Vocal individuality provides a crucial prerequisite for individual recognition (Pollard and Blumstein 2012; Tibbetts and Dale 2007). Specifically, vocal signals encoding information of individual identity can be utilized to make decisions about whether to approach, avoid, or ignore particular individuals (Chapman and Weary 1990) and thus help to mediate social relationships within and between groups (Bolt and Tennenhouse 2017). For example, whinny calls of spider monkeys (*Ateles geoffroyi*) exhibit individual distinctiveness and are used to maintain appropriate cohesion and spacing when group members forage outside of each other’s visual range (Ramos-Fernández 2005). African penguins (*Spheniscus demersus*) produce contact calls containing information about individual identity, which are used by isolated individuals to rejoin companions (Favaro et al. 2015). In addition, vocal individuality is also essential for kin selection such as in the context of parental investment (*Callorhinus ursinus*: Charrier et al. 2003; *Papio cynocephalus ursinus*: Rendall et al. 2000), and for sexual selection, which involves male–male competition and female choice (*Cervus elaphus*: McComb 1991).

It has been suggested that individuality of acoustic signals is more important for species living in large and complex social systems, which include more interacting individuals, more diverse interactions, and/or more social structural levels (Freeberg et al. 2012; Pollard and Blumstein 2011, 2012; Tibbetts and Dale 2007). For example, some call types emitted by species with fission–fusion social dynamics exhibit clear individuality, such as contact calls of African elephants (McComb et al. 2003), whistles of bottlenose dolphins (*Tursiops truncatus*) (Janik and Slater 1998; Tyack 2000), and grunts of chacma baboons (*Papio cynocephalus ursinus*) (Owren et al. 1997; Rendall 2003). A comparative study of eight sciurid rodent species has shown that group size facilitates the evolution of individuality in alarm calls (Pollard and Blumstein 2011). However, evidence for vocal individuality in Asian colobine primates living in large and multilevel societies is still lacking.

The source-filter theory of vocal production, originated from human voice studies (Titze 1994), states that the fundamental frequencies and formant frequencies vary among individuals due to the differences in the length and shape of the callers’ vocal apparatus (Reby and McComb 2003), and one or both may provide robust individual distinctiveness. The source-filter theory has been widely applied to vocal studies in nonhuman animals (Charlton et al. 2010, 2017; Taylor and Reby 2010). For example, the fundamental frequencies of coo calls encode information of individual identity in Japanese macaques (*Macaca fuscata*) (Ceugniet and Izumi 2004). Formant frequencies play an important role in the individuality in contact calls of African elephants (McComb et al. 2003) and grunt calls of red-bellied lemurs (*Eulemur rubriventer*) (Gamba et al. 2012). Both fundamental frequency and formant parameters encode information of individual identity in contact calls of African penguins (Favaro et al. 2015).

The golden snub-nosed monkey (*Rhinopithecus roxellana*), a colobine species endemic to China, inhabits temperate forests in mountainous areas at high altitudes of 1000–4100 m and lives in large groups varying from 80 to more than 400 individuals (Kirkpatrick and Grueter 2010). Its social organization is described as a multilevel society, which comprises one breeding band consisting of several one-male multi-female units (OMUs) and one (occasionally more than one) peripherally attached all-male unit (AMU) (Qi et al. 2014; Yao et al. 2011; Zhang et al. 2006). The OMUs of the breeding band coordinate their activities on a day-to-day basis, while each of them is a relatively independent social entity maintained mainly by matrilineal kin-bonds (Wang et al. 2013; Zhang et al. 2012). The AMU comprises former OMU resident males who have been replaced, and subadult and juvenile males waiting for opportunities to take over the resident positions or to emigrate to other groups (Qi et al. 2017; Yao et al. 2011). Inter-individual interactions within and between units include both competitive and cooperative elements (Liu et al. 2016; Wada et al. 2015; Xiang et al. 2014; Zhang et al. 2010). The ecological and social settings of this primate (forest habitats with limited visibility and large groups with a multilevel structure) are expected to be conducive to the evolution of high levels of vocal individuality.

However, to date, it is not yet known whether and how vocal signals of *R. roxellana* (and the genus of snub-nosed monkeys in general) can convey information of individual identity. Coo calls are one of the most frequently occurring call types in adult *R. roxellana* and likely function to maintain contact in various contexts including group movement, foraging, and resting (Fan et al. 2018). The spectrogram of coo calls is characterized by few frequency modulations and rich harmonic patterns (Fan et al. 2018), and the dense harmonic structure should highlight the formants, making these vocalizations well suited for individual discrimination (Charlton et al. 2009b; Owren and Rendall 2001). Here, based on the source-filter theory, we investigated the individuality in coo calls of adult male *R. roxellana*. We chose adult males as our study subjects, because they play an important role in the maintenance of social cohesion and spacing at both levels within and between units (Huang et al. 2017; Qi et al. 2017; Xiang et al. 2014). We first examined whether coo calls had a sufficient degree of individual distinctiveness that would permit discrimination among callers. We then examined and identified the key acoustic parameters determining the distinctiveness among different individuals. The findings of this study will improve our understanding of social cognition in species living in large and multilevel societies.

## Methods

### Study site and subjects

This study was carried out at the Dalongtan Conservation Station (DCS) and the Golden Snub-nosed Monkey Reproduction Center (GRC) in Shennongjia National Park, Hubei Province, China. To facilitate ecotourism and research, a monkey group at DCS has been habituated and provisioned since 2006 (Yao et al. 2011). Food items including lichens, pine seeds, apples, carrots, oranges, and peaches are provisioned two or three times a day. When not provisioned, the monkeys range freely within an area of approximately 9 km^2^, characterized by a deciduous broadleaf and evergreen conifer mixed forest. We can identify all adult individuals based on their physical features (e.g., body size, hair coloration, scar, and face shape) in proximity (0.5–10 m). During the period from April to October 2016, the monkey group was composed of five OMUs (containing one adult male in each: GE, HH, NN, XB, and XZ) and one AMU (containing two adult males: DD and HT). In October 2016, an OMU male, NN, was replaced by an AMU male, DD. After being taken over, NN moved to the AMU and then emigrated entirely from the monkey group in November 2016. In December 2016, DD transferred back to the AMU since his unit members joined the OMU of XZ voluntarily. From that time to the end of this study, no change occurred in the unit memberships of adult males.

GRC, about 1200 m away from DCS, is responsible for rescuing and breeding injured monkeys from the wild. During the study period, an adult male, DW, was rescued and kept in captivity at GRC. Food items fed to the monkey are the same as those to the DCS group.

A total of seven adult males were selected as our study subjects, six (DD, GE, HH, NN, XB, and XZ) from DCS and one (DW) from GRC. The adult male from the DCS group, HT, was excluded because of the difficulties associated with approaching him to collect ample vocalization samples.

### Vocalization recordings

We recorded vocalizations outside of the provisioning times and when there were not excessive human disturbances during the period from April to October 2016, April to July 2017, and September to October 2017. Vocalizations were collected at a sampling rate of 44.1 kHz (16 bit) using a Tascam DR44-WL digital recorder connected to a Sennheiser ME 66 directional microphone at distances within 10 m to the monkeys. For the DCS group, we selected one adult male as the subject on an observation day (08:00–18:00) and recorded his coo calls using 5-min focal animal sampling (Fan et al. 2018). We then rotated to another on the next day. Occasionally, we recorded calls of non-focal adult males opportunistically to increase the total amount of coo call samples using ad libitum sampling. For the adult male at GRC, we also used 5-min focal animal sampling to collect coo call samples. We recorded coo calls of GE over 15 days, HH over 21 days, NN over 17 days, XB over 24 days, XZ over 22 days, DD over 14 days, and DW over 7 days. The vocalization data were uploaded to a laptop computer for storage and analysis.

This study complied with the animal protection laws of the People’s Republic of China and was approved by the Committee of Animal Welfare and Ethic of the Beijing Normal University, the University of Chinese Academy of Sciences, and Shennongjia National Park. We made efforts to minimize potential disturbances to the monkeys during vocalization recording.

### Acoustic parameter measurements

**Fig. 1:**
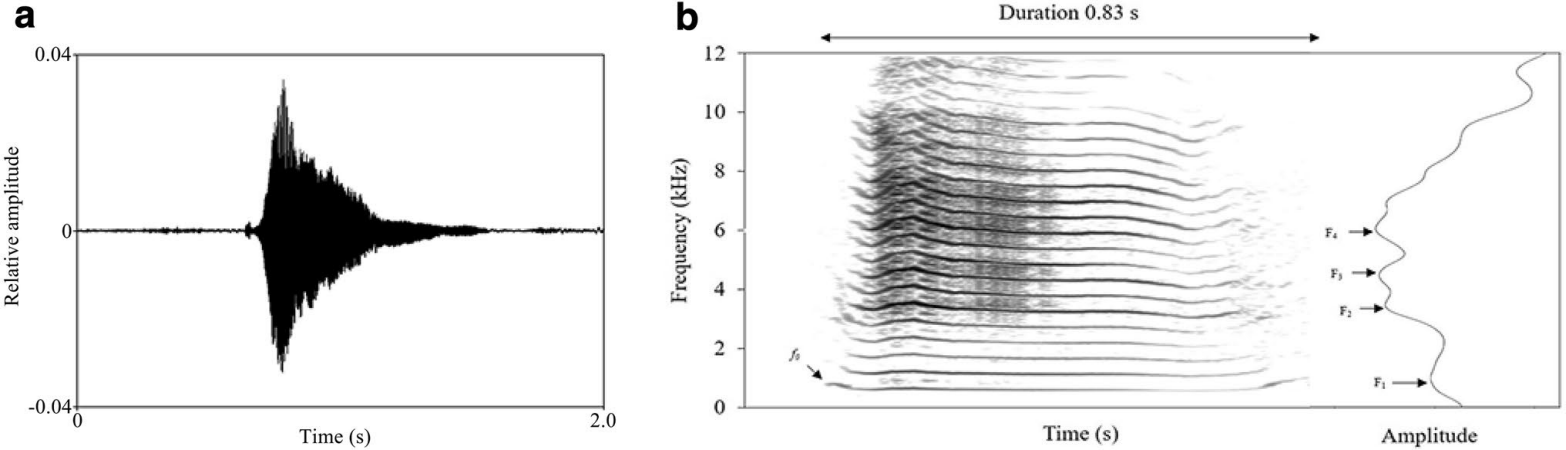
The waveform of a coo call from an adult male *R. roxellana* (**a**); Spectrogram (Gaussian window shape, view range = 0 − 12000 Hz, window length = 0.03 s, dynamic range = 70 dB, time step = 0.002 s, frequency step = 20 Hz) and LPC spectrum (Cepstral smoothing: 1200 Hz) of the coo call showing *f*_0_ and formants (*F*_1_–*F*_4_) (**b**)

We used Adobe Audition CS6 (Adobe, USA) and Praat package 5.3.72 (P. Boersma and D. Weenink, University of Amsterdam, the Netherlands) for acoustic analyses. All vocalizations were standardized in Adobe Audition CS6. We then carried out visual and acoustical inspection of each coo call with narrow-band spectrograms generated by the Praat sound editor window (Gaussian window shape, view range = 0–12,000 Hz, window length = 0.03 s, dynamic range = 70 dB; Fig. 1. We excluded from further analysis poor-quality recordings with excessive background noise such as bird and stream sounds, and those that overlapped with other calls. For each high quality recording selected, we measured a series of acoustic parameters, including temporal (call duration), source-related (fundamental frequency: *f*_0_), and filter-related features (formant), and mean harmonics-to-noise ratio (HNR). We extracted the *f*_0_ contour of recordings using a cross-correlation method [Sound: To Pitch (cc) command; time step = 0.01 s, pitch floor = 75 Hz, pitch ceiling = 1200 Hz]. We measured temporal and source-related parameters including call duration, and the mean (mean *f*_0_), start (start *f*_0_), end (end *f*_0_), minimum (min *f*_0_), maximum (max *f*_0_), and standard deviation (SD *f*_0_) of fundamental frequency values from each extracted *f*_0_ contour. We calculated the range of fundamental frequency (range *f*_0_) as max *f*_0_ minus min *f*_0_. To measure formant parameters, we extracted the first four mean formants (*F*_1_–*F*_4_) of each recording using a Linear Predictive Coding analysis [Sound: To Formant (burg) command; time step = 0.01 s, maximum number of formants = 5, maximum formant = 8000 Hz]. We then used the method described by Reby and McComb (2003) to calculate the value of formant dispersion (Δ*F*). Finally, we measured the HNR value of each recording using the “To Harmonicity (cc) command” (time step = 0.01 s, minimum pitch = 75 Hz, silence threshold = 0.1, and periods per window = 1).

### Statistical analysis

We first calculated within-individual (*CV*_w_) and between-individual (*CV*_b_) coefficients of variation for each acoustic parameter as follows: *CV* = 100 (1 + 1/4*n*) (SD/$\bar {x}$) (Robisson et al. 1993). In this formula, *n* represents the sample size of vocalizations, *SD* the standard deviation of the sample, and $\bar {x}$ the mean value. We calculated the potential for individual identity coding (PIC) using the ratio of the *CV*_b_ to the mean *CV*_w_ for all individuals (Gamba et al. 2012). For each acoustic parameter measured, a PIC value more than 1 indicates that this parameter has the potential for individual discrimination because of the lower variability within individuals than between individuals (Robisson et al. 1993). Furthermore, we performed a Kruskal–Wallis test to investigate which acoustic parameter was different among individuals. If the test yielded a significant result for a parameter, we carried out pairwise comparisons using Mann–Whitney *U* tests.

To quantify the individual distinctiveness of coo calls, we performed a principal component analysis (PCA) and subsequently a discriminant function analysis (DFA). PCA allowed us to obtain a reduced number of orthogonal variables (principal components: PCs) that accounted for the most amount of variance in the data set. We retained the PCs with eigenvalues greater than 0.6 (Kaiser’s criterion) using a varimax rotation method to improve component interpretation (Vannoni and Mcelligott 2007). These PCs were tested for normality (Kolmogorov–Smirnov test), and then used as input variables in the subsequent DFA. Based on the discriminant functions combined by the predictor variables that best describe the differences among groups, DFA assigns each vocalization to its appropriate group (correct) or another group (incorrect). Because the number of calls per individual was unbalanced, classification coefficients were adjusted according to the observed group sizes. For cross validation, we used the leave-one-out classification method, in which each case was classified by the functions derived from all cases except that one. All data were analysed with SPSS 21.0, and the tests were two-tailed with a significance level of 0.05 except the Mann–Whitney *U* tests, in which we used the Bonferroni adjusted significance level of 0.05/21 = 0.002.

## Results

We recorded a total of 721 vocalization samples during the study period and selected 162 high quality recordings for further analysis (Table S1). We found that the *CV*_b_ value of each acoustic parameter was higher than the mean *CV*_w_ value, and thus all PIC values were greater than 1 (Table 1). The Kruskal–Wallis tests showed that each of all parameters was significantly different among individuals (Table 2). However, the pairwise comparisons failed to detect any parameter that was different between all pairs.

**Table 1:** The coefficients of variation within (*CV*_w_) and between individuals (*CV*_b_), and the potential for individual identity coding (PIC) for each acoustic parameter of coo calls from adult male *R. roxellana*

Parameter	Mean *CV*_w_ (%)	*CV*_b_ (%)	PIC
Duration	15.80	18.67	1.18
Mean *f*_0_	7.39	9.98	1.35
SD *f*_0_	33.33	36.62	1.10
Max *f*_0_	9.89	13.79	1.39
Min *f*_0_	22.42	29.59	1.32
Range *f*_0_	31.72	35.08	1.11
Start *f*_0_	12.88	17.08	1.33
End *f*_0_	25.55	30.38	1.19
HNR	18.79	28.10	1.50
*F*_1_	8.52	10.08	1.18
*F*_2_	3.56	5.32	1.49
*F*_3_	2.72	5.24	1.92
*F*_4_	3.16	3.64	1.15
Δ*F*	4.31	4.96	1.15

**Table 2:** Inter-individual comparisons for each acoustic parameter of coo calls from adult male *R. roxellana*

Parameter	Kruskal–Wallis test		Pairs for which differences were detected based on Mann–Whitney *U* tests*
	*χ* ^2^	*P* value	
Duration	52.71	< 0.001	DD-GE, DD-XZ, DW-GE, GE-HH, GE-NN, GE-XB, HH-XB, HH-XZ, NN-XB, NN-XZ
Mean *f*_0_	74.34	< 0.001	DD-DW, DD-GE, DD-HH, DD-NN, DD-XB, DD-XZ, DW-HH, DW-XB, GE-HH, GE-XB, HH-XZ, NN-XZ XB-XZ
SD *f*_0_	23.65	0.001	DW-GE, DW-XZ
Max *f*_0_	40.02	< 0.001	DD-DW, DD-GE, DD-NN, DD-XB, DD-XZ, DW-NN, DW-XB, GE-NN, NN-XZ, XB-XZ
Min *f*_0_	59.62	< 0.001	DD-DW, DD-GE, DD-HH, DD-NN, DD-XB, DD-XZ, DW-XB, GE-HH, GE-NN, GE-XB, HH-XZ, NN-XZ XB-XZ
Range *f*_0_	13.88	0.031	GE-XB
Start *f*_0_	60.04	< 0.001	DD-GE, DD-NN, DD-XZ, DW-GE, DW-NN, DW-XZ, GE-HH, HH-NN, HH-XB, HH-XZ, XB-XZ
End *f*_0_	45.16	< 0.001	DD-GE, DD-XB, DD-XZ, DW-NN, GE-NN, NN-XB, NN-XZ
HNR	69.65	< 0.001	DD-GE, DD-XB, DD-XZ, DW-GE, DW-XZ, GE-HH, GE-NN, GE-XB, HH-NN, NN-XB, NN-XZ
*F*_1_	47.39	< 0.001	DD-DW, DW-GE, DW-NN, DW-XB, DW-XZ, HH-XB, HH-XZ
*F*_2_	76.88	< 0.001	DD-GE, DD-HH, DD-NN, DD-XB, DD-XZ, DW-GE, DW-HH, DW-XB, DW-XZ, GE-NN, HH-NN, NN-XB NN-XZ
*F*_3_	95.48	< 0.001	DD-DW, DD-NN, DW-GE, DW-HH, DW-NN, DW-XB, DW-XZ, GE-HH, GE-NN, HH-NN, HH-XB, NN-XB, NN-XZ
*F*_4_	68.34	< 0.001	DD-DW, DD-GE, DD-NN, DW-HH, DW-XB, DW-XZ, GE-HH, GE-XB, GE-XZ, HH-NN, NN-XB, NN-XZ
Δ*F*	64.49	< 0.001	DD-DW, DD-GE, DD-NN, DW-HH, DW-XB, DW-XZ, GE-HH, GE-XB, GE-XZ, HH-NN, NN-XB, NN-XZ

*Adjusted significance level of 0.05/21 = 0.002

The first seven PCs explained 91.7% of the total variance in the data set (Table S2). Based on the seven PCs, DFA correctly assigned 80.2% of coo calls (Table 3). The classification accuracy of cross-validation was 67.3%, which was better than the 14.3% expected by chance (binomial test, *P* < 0.001). DFA generated six canonical discriminant functions, and the first three had eigenvalues > 0.5 (Function 1: 2.4, Function 2: 1.7, and Function 3: 0.6) and cumulatively explained 95.0% of the variance (Table S3). Function 1 explained 48.5% of the variance and was primarily related to PC3 and PC5. PC3 was mainly associated with Range *f*_0_, Min *f*_0_ and SD *f*_0_, while PC5 with *F*_1_ and *F*_4_. Function 2 explained 35.0% of the variance and was primarily associated with PC4, which was most strongly related to *F*_1_. Function 3 explained 11.5% of the variance and was mainly related to PC7, which was primarily associated with call duration.

**Table 3:** The classification (cross-validation) of discriminant function analysis for seven individuals of adult male *R. roxellana*

Individual	Predicted classification							
	DD	GE	HH	NN	XB	XZ	DW	Total
DD	7 (6)	0 (0)	0 (1)	1 (1)	4 (4)	0 (0)	0 (0)	12
GE	0 (0)	12 (11)	0 (0)	0 (0)	0 (1)	4 (4)	0 (0)	16
HH	3 (4)	0 (0)	25 (20)	0 (0)	4 (5)	1 (2)	0 (2)	33
NN	0 (1)	0 (0)	0 (0)	21 (18)	0 (0)	0 (1)	2 (3)	23
XB	0 (1)	0 (0)	2 (2)	0 (0)	33 (32)	4 (4)	1 (1)	40
XZ	0 (0)	2 (4)	1 (2)	1 (1)	1 (3)	18 (12)	1 (2)	24
DW	0 (0)	0 (1)	0 (3)	0 (0)	0 (0)	0 (0)	14 (10)	14

## Discussion

In the present study, we found that coo calls of adult male *R. roxellana* living in a large and multilevel social system could encode information of individual identity. Furthermore, all acoustic parameters analyzed in our study complementarily contributed to the differences in coo calls among individuals. This result supports the notion that subtle combinations of different acoustic features make up the call characteristics of an individual caller (Epsmark 1975). Similar findings that multiple parameters complementarily contribute to vocal individuality have also been reported in several other mammals and birds (*Papio hamadryas ursinus*: Rendall 2003; *Presbytis thomasi*: Wich et al. 2003; *Dama dama*: Vannoni and McElligott 2007; *Pan troglodytes*: Levréro and Mathevon 2013; *Spheniscus demersus*: Favaro et al. 2015; *Bos taurus*: Torre et al. 2015).

The acoustic parameters that contributed most to individuality were duration (temporal parameter), Range *f*_0_, Min *f*_0_, SD *f*_0_ (source-related parameters), and *F*_1_, *F*_4_ (filter-related parameters). This result suggests that three different parts of the respiratory apparatus, i.e., the lungs, vocal folds, and vocal tract, played important roles in producing and shaping the inter-individual differences in coo calls of adult male *R. roxellana*. Duration of acoustic waveform is determined by the airflows modulated by the chest muscles and the vital capacity of callers (Favaro et al. 2015). Therefore, call duration exhibits relatively stabilized variability within individuals (Favaro et al. 2015; Haimoff and Tilson 1985) and has the capacity to convey acoustically information about individual identity.

Differences in the source-related parameters are mainly determined by the length and stiffness (tension) of the vocal folds (Titze 1994). In general, the shorter and stiffer the vocal folds are, the higher the frequency is. Range *f*_0_ represents the difference between max *f*_0_ and min *f*_0_, while min *f*_0_ reflects the minimum rate of vibration of the vocal folds, which is physiologically constrained by its length (Titze 1994; Fitch 1997). SD *f*_0_, the standard deviation of fundamental frequency values, can be related to the stiffness of the vocal folds (Charlton et al. 2010). These characteristics of vocal folds may show some differences among individual callers of adult male *R. roxellana* (Charlton et al. 2009b). Individual distinctiveness in the source-related parameters of vocalizations have also been found in other animals, such as grunts of Guinea baboons (*Papio papio*) (Owren et al. 1997) and coo calls of Japanese macaques (Ceugniet and Izumi 2004).

Unlike the source-related features, the filter-related features of acoustic signals are determined by the shape and length of the vocal tract (Titze 1994). Specifically, lower formants are determined by the shape of the vocal tract, while higher formants are determined by the length (Reby and McComb 2003). The structure of the vocal tract is strongly related to body size (Fitch 1997; Reby and Mccomb 2003; Torre et al. 2015), and thus individual variation in formants is likely to reflect the differences in body size among callers (Pfefferle and Fischer 2006). In our study, both lower (*F*_1_) and higher (*F*_4_) formants were among the parameters most strongly related to individuality, suggesting that the shape and length of the vocal tract may vary among individuals of adult male *R. roxellana*. Several other studies have reported that the filter-related features are indicators of vocal individuality (Reby et al. 2006; Soltis et al. 2005), such as bleat calls of giant pandas (*Ailuropoda melanoleuca*) (Charlton et al. 2009b) and grunt calls of red-bellied lemurs (Gamba et al. 2012).

Interestingly, we found that HNR of coo calls had the potential for individual discrimination in adult male *R. roxellana*. HNR represents the ratio of harmonics to noise in spectrum resulting from turbulent airflows generated at the glottis during phonation (Hillenbrand 1987). Previous studies in humans have shown that the HNR values in elderly women are lower than those of juveniles and prime adults, suggesting that HNR may be a sensitive index of body aging, such as the ossification of cartilage and the degeneration of muscles and connective tissues in the larynx and vocal tract (Brown et al. 1996; Ferrand 2002). Similar findings that vocal structure can reflect age information have also been reported in some nonhuman animals, such as bleats of giant pandas (Charlton et al. 2009a) and loud calls of male chacma baboons (Fischer et al. 2004). Thus, HNR differences in coo calls among individuals of adult male *R. roxellana* may be a by-product of differences in age. Age information in vocal signals may advertise the callers’ physical quality indirectly (Fischer et al. 2004), which may further affect social relationships among different individuals.

Contact calls of social animals, such as coos of *R. roxellana*, serve as affiliative vocal signals that have evolved to coordinate group movement and establish and maintain social relationships with conspecifics (Bolt and Tennenhouse 2017; Kondo and Watanabe 2009). There is accumulating evidence that contact calls can be used for individual recognition (Sharpe et al. 2013), which is a critical precondition for successfully navigating a large and complex social landscape (Pollard and Blumstein 2012; Tibbetts and Dale 2007). *Rhinopithecus roxellana* lives in large and multilevel societies composed of several socio-spatially distinct units (Qi et al. 2014, 2017). The large group size and social complexity could constitute a strong selection force for the evolution of individuality in coo calls, facilitating individual discrimination (Pollard and Blumstein 2012), if individuals of a social unit are able or motivated to interact closely with or keep track of those of other units. While previous studies of some primates with multilevel societies noted the absence of such an ability or motivation (Bergman 2010; Maciej et al. 2013), studies of *R. roxellana* have shown that the social units of a group coordinate their activities on a daily basis (Liu et al. 2016; Wada et al. 2015; Zhang et al. 2010) and that the animals engage in particularly significant interactive events among units (Qi et al. 2017). For example, the resident males have been observed to collectively defend their OMUs against the bachelor males of the AMU (Huang et al. 2017; Xiang et al. 2014). The adult females of an OMU copulate with the males of other units and sire offspring (Guo et al. 2010; Zhao et al. 2005).

Although the capability of individual discrimination via coo calls in *R. roxellana* needs to be verified by playback experiments in further studies, the concurrent contexts of these vocalizations suggested that receivers could be able to recognize particular callers. Specifically, we observed that the resident males uttered coo calls towards the direction of their unit members that were out of sight during unit/group movement in the dense forest. Sometimes, the unit members responded vocally to these vocalizations (Fan et al. 2018). The resident males would continuously emit coo calls if their unit members did not catch up. Individual discrimination via vocal signals would allow the animals living in forest habitats to make adaptive decisions with regards to which individuals (and thus units/groups) to approach, avoid or ignore (Chapman and Weary 1990; Delgado 2007). For example, adult females may benefit from being able to recognize particular adult males based on vocal cues by reducing the risk of infanticide (Yao et al. 2016), as observed in Thomas langurs (*Presbytis thomasi*) (Wich 2002).

It is worth noting that the correct classification rate of DFA was not very high (67.3% by cross validation vs. 14.3% expected by chance), especially with respect to the large and complex social system of *R. roxellana*. It is very likely that the relatively small number of study subjects reduced the discriminant rate (Pfefferle et al. 2016). Alternatively, the vocalization samples occurred in various contexts, and the context-related variation in the acoustic structure may have partially masked the differences among individuals (Wich et al. 2003). Future studies are needed to address how vocal signals convey individuality information of the callers and contextual information of the calls.

## Electronic supplementary material

Below is the link to the electronic supplementary material.

Supplementary material 1 (PDF 272 KB)
