# Supplementary material for: Individuality in coo calls of adult male golden snub-nosed monkeys (Rhinopithecus roxellana) living in a multilevel society
Source: Anim Cogn. 2018 Nov 20;22(1):71–9. doi: 10.1007/s10071-018-1222-y (PMC6326966; doi:10.1007/s10071-018-1222-y)
Supplement: Supplementary file 1 — Supplementary material 1 (PDF 272 KB) [file 10071_2018_1222_MOESM1_ESM.pdf]

ANIMAL COGNITION

Individuality in coo calls of adult male golden snub-nosed monkeys (*Rhinopithecus roxellana*) living in a multilevel society

Penglai Fan, Ruoshuang Liu, Cyril C. Grueter, Fang Li, Feng Wu, Tianpeng Huang, Hui Yao, Dingzhen Liu*, Xuecong Liu*

* Dingzhen Liu: Key Laboratory of Biodiversity Science and Ecological Engineering (Ministry of Education), College of Life Sciences, Beijing Normal University, Beijing 100875, China; dzliu@bnu.edu.cn
* Xuecong Liu: College of Life Sciences, University of Chinese Academy of Sciences, Beijing 100049, China; xuecongliu@ucas.ac.cn

Table S1 Acoustic parameters (mean ± SD) of coo calls collected from seven individuals of adult male *R. roxellana*

Parameter	Individual (Number of all vocalizations, number of vocalizations used for acoustic analysis)						
	DD (45, 12)	GE (42, 16)	HH (150, 33)	NN (82, 23)	XB (207, 40)	XZ (149, 24)	DW (46, 14)
Duration (s)	0.66 ± 0.11	0.91 ± 0.09	0.68 ± 0.11	0.69 ± 0.07	0.77 ± 0.07	0.84 ± 0.18	0.77 ± 0.19
Mean f_0 (Hz)	427.2 ± 25.9	522.8 ± 28.7	482.0 ± 52.3	491.8 ± 42.5	464.1 ± 34.1	526.5 ± 19.6	530.8 ± 40.8
SD f_0 (Hz)	86.9 ± 16.3	70.3 ± 19.7	87.5 ± 40.1	89.2 ± 32.3	80.6 ± 21.8	63.8 ± 28.2	102.5 ± 22.0
Max f_0 (Hz)	559.9 ± 63.3	690.4 ± 51.6	654.9 ± 118.5	635.5 ± 52.2	639.8 ± 85.7	717.9 ± 81.2	713.4 ± 27.1
Min f_0 (Hz)	232.5 ± 26.3	421.5 ± 56.0	328.8 ± 80.0	305.6 ± 89.1	284.9 ± 84.1	417.2 ± 94.2	342.7 ± 86.7
Range f_0 (Hz)	327.5 ± 73.4	269.0 ± 59.1	326.1 ± 152.1	329.9 ± 95.9	354.9 ± 103.1	300.7 ± 134.6	370.6 ± 94.5
Start f_0 (Hz)	513.2 ± 51.8	628.5 ± 53.8	491.4 ± 90.8	592.2 ± 92.3	563.9 ± 87.3	656.8 ± 73.3	522.0 ± 50.5
End f_0 (Hz)	407.9 ± 100.6	619.5 ± 107.3	497.0 ± 196.5	383.6 ± 101.2	542.3 ± 134.0	580.1 ± 144.5	522.5 ± 91.8
HNR	7.7 ± 1.7	14.6 ± 1.7	10.3 ± 2.8	8.0 ± 1.1	10.7 ± 2.4	12.4 ± 2.6	8.8 ± 1.8
F_1 (Hz)	1003.4 ± 69.1	996.9 ± 72.2	954.8 ± 77.8	1024.8 ± 122.2	1048.9 ± 99.4	1038.9 ± 93.1	888.8 ± 34.3
F_2 (Hz)	3251.9 ± 110.8	3094.3 ± 107.6	3117.2 ± 119.1	3414.3 ± 114.0	3098.8 ± 131.1	3091.8 ± 150.0	3313.4 ± 78.2
F_3 (Hz)	4237.8 ± 199.9	4168.6 ± 96.3	4315.5 ± 121.2	4652.2 ± 75.5	4155.4 ± 155.2	4191.7 ± 172.1	4568.1 ± 73.8
F_4 (Hz)	5786.7 ± 349.3	6147.2 ± 239.7	5806.5 ± 141.6	6068.7 ± 132.8	5860.6 ± 120.9	5888.0 ± 112.1	6161.2 ± 192.3
ΔF	1594.5 ± 116.8	1716.7 ± 73.8	1617.2 ± 59.3	1681.3 ± 62.5	1603.9 ± 45.8	1616.3 ± 52.4	1757.5 ± 66.9

Table S2 Rotated factor loadings of the acoustic parameters on first seven principal components (eigenvalues > 0.6)

Parameters	Principal components						
	1	2	3	4	5	6	7
Duration	0.61	-0.06	-0.05	-0.38	0.27	0.31	0.44
Mean f_0	0.75	0.10	0.40	0.28	-0.25	0.00	0.15
SD f_0	0.09	0.69	-0.65	-0.04	-0.06	-0.01	0.03
Max f_0	0.83	0.35	-0.27	0.18	-0.11	-0.05	-0.07
Min f_0	0.55	-0.36	0.65	0.23	-0.21	0.03	-0.03
Range f_0	0.18	0.58	-0.77	-0.05	0.09	-0.07	-0.03
Start f_0	0.62	0.03	0.01	0.31	0.35	-0.57	0.03
End f_0	0.78	0.08	-0.29	0.11	-0.23	0.34	-0.20
HNR	0.43	-0.52	-0.10	-0.39	0.21	-0.15	0.23
F_1	-0.04	-0.28	-0.14	0.62	0.63	0.30	-0.10
F_2	-0.36	0.65	0.25	0.31	0.04	0.10	0.38
F_3	-0.23	0.76	0.37	0.27	-0.05	-0.03	0.18
F_4	0.21	0.57	0.56	-0.24	0.41	0.10	-0.27
ΔF	0.21	0.62	0.55	-0.47	0.11	-0.03	-0.19
Eigenvalue	3.41	3.12	2.61	1.40	1.00	0.68	0.61
Variance explained (%)	24.35	22.31	18.62	10.01	7.14	4.87	4.38

Table S3 Structure matrix of discriminant function analysis with the first three standardized canonical discriminant functions (eigenvalues > 0.5)

PC scores	Discriminant functions		
	1	2	3
PC1	-0.44	-0.37	0.05
PC2	0.47	0.32	-0.51
PC3	-0.88	0.58	0.14
PC4	0.04	0.74	-0.23
PC5	0.74	0.52	0.01
PC6	0.38	0.65	0.45
PC7	0.36	-0.12	0.80
Eigenvalue	2.42	1.75	0.57
Variance explained (%)	48.5	35.0	11.5
